# Content of ^13^С and ^15^N Isotopes in Bone Collagen of Geographical, Age, and Sex Groups of the Ural Cave Bear (Mammalia, Carnivora, Ursidae, *Ursus (Spelaearctos) kanivetz* Verestchagin, 1973)

**DOI:** 10.1134/S001249662370076X

**Published:** 2023-11-11

**Authors:** P. A. Kosintsev, K. Yu. Konovalova, G. V. Simonova

**Affiliations:** 1 Institute of Plant and Animal Ecology, Ural Branch, Russian Academy of Sciences, Yekaterinburg, Russia; 2 Institute of Monitoring of Climatic and Ecological Systems Siberian Branch Russian Academy of Sciences, Tomsk, Russia

**Keywords:** *Ursus kanivetz*, Ural cave bear, Late Pleistocene, the Urals, stable isotope, ^13^C, ^15^N, collagen

## Abstract

Data on the content of ^13^C and ^15^N isotopes in the collagen of bones of the Ural cave bear (*Ursus* (*S*.) *kanivetz* Verestchagin, 1973) from the North and Middle Urals were analyzed. The bones date from the first half of MIS 3. The bones of newborn individuals, individuals aged 1 year, males and females aged 2, 3, and 4 years, and older than 4 years were studied. Differences in δ^13^С values between age, sex, and geographical samples are not significant. With age, the value of δ^15^N significantly decreases, which is associated with weaning from milk nutrition to independent nutrition. The proportion of meat food in the diet of adult bears in the Middle Urals was higher than in the diet of adult bears in the North Urals. There are no noticeable differences in isotope signatures between males and females of different ages. The large cave bears of the Urals and Europe had a similar type of diet.

Analysis of the content of ^13^C and ^15^N isotopes in the tissues of living organisms is widely used to reconstruct the habitat and ecology of species in the past and present. It allows characteristics of the nutrition of the species and its position in the trophic chain to be evaluated. The study of isotopes is especially important for extinct species. One of the largest data sets on the isotopic composition of carbon and nitrogen in bone collagen was obtained for large cave bears (*Ursus* (*Spelaearctos*) *spelaeus* s.l.) of Western and Central Europe [1–7]. Data on the content of ^13^C and ^15^N isotopes in the collagen of the bones of large cave bears in Eastern Europe and the Urals are extremely scarce [8].

Analysis of morphological data and nuclear DNA showed that one species of large cave bear lived in Eastern Europe and the Urals in the late Pleistocene, i.e., the Ural cave bear (*Ursus* (*S.*) *kanivetz* Verestchagin, 1973) [9, 10]. Three species lived in Central Europe: *U.* (*S*.) *spelaeus* Rosenmuller 1794, *U.* (*S.*) *eremus* Rabeder, Hofreiter, Nagel et Withalm, 2004, and *U.* (*S.*) *kanivetz* Verestchagin, 1973. In Western Europe, there were two species of large cave bears: *Ursus* (*S.*) *spelaeus* Rosenmuller 1794 and *U.* (*S*.) *eremus* Rabeder, Hofreiter, Nagel et Withalm, 2004 [10, 11].

The content of ^13^C and ^15^N isotopes in the collagen of the bones of the Ural cave bear from the Tayn Cave in the Middle Urals [8] and the Medvezhiya Cave in the North Urals was studied. The selection from the Cave of Secrets has been supplemented with new specimens. Bear Cave (62°05′ N, 58°05′ E) is of karst origin, horizontal type of structure, length 480 m and height above sea level 280 m [12]. This is the northeastern edge of the range (*Ursus* (*S.*) *spelaeus* s.l.) [9]. More than 3000 bones of a large cave bear were found in the cave sediments, among which there are remains of all age groups from newborns to old individuals. This is a typical “graveyard” for cave bears, where the animals died during hibernation. Radiocarbon (AMS) dates were obtained from the cave bear bones: >48 600 BP, no.? [13]; 42 000 ± 450 BP, OxA-19 608; 45 150 ± 600 BP, OxA-19 568 [10], which corresponds to the first half of Late Pleistocene marine isotope stage 3 (MIS 3). The Tain and Medvezhya caves have the same taphonomic type (“cemetery”), the accumulation of bones occurred in one period—the first half of MIS 3 (57 000–40 000 years ago).

For analysis, 45 humeri, eight radii, and one tibia were taken. Determination of the sex and age of the individuals to which the bones belonged was determined based on analysis of their size and the condition of the epiphyses (accreted or not accreted) [14–17]. Among them there are bones of males and females aged 2+, 3+, and 4+ years (subadults) and older than 4+ years (>4+, adults, adults) (Table 1). The sex of individuals aged 0+ and 1+ years is not determined. Each bone belongs to a separate individual. Individuals aged 0+ have only milk feeding, individuals aged 1+ have a mixed type of feeding (milk and independent), from age 2+ animals switch to independent feeding [18].

**Table 1.** Values of δ^13^С and δ^15^N (‰) and standard deviation (sd) in collagen of the bones of the Ural cave bear (*U.* (*S*.) *kanivetz*) of the North and Middle Urals

Sex	Age	*n*	δ ^13^C_min_	δ ^13^C_max_	δ ^13^C_av_ ± sd	δ ^15^N_min_	δ ^15^N_max_	δ ^15^N_av_ ± sd
		North Urals						
♂	>4+	9	–23.6	–21.2	–22.3±0.84	2.4	5.4	3.9±1.04
♀		5	–23.4	–21.8	–22.6±0.58	2.8	4.9	3.8±0.93
♂	3+, 4+	4	–22.2	–21.7	–21.9±0.21	3.9	5.8	4.8±0.84
♀		3	–23.6	–21.7	–22.4±1.01	3.4	4.2	3.9±0.44
♂	2+	4	–23.1	–21.9	–22.5±0.61	4.3	6.6	5.2±0.98
♀		4	–22.1	–20.2	–21.0±0.94	4.6	11.3	7.1±3.08
?	1+	23	–22.9	–21.4	–22.4±0.35	5.0	8.4	6.7±0.86
?	0+	2	–23.9	–22.4	–23.2±1.08	8.3	9.0	8.7±0.52
		Middle Urals						
♂	>4+	5	–22.1	–21.3	–21.7±0.37	3.2	4.9	4.1±0.68
♀		2	–22.2	–21.8	–22.0±0.26	4.6	4.6	4.6
♂	3+, 4+	3	–22.5	–22.1	22.2±0.23	3.2	4.0	3.7±0.42
♀		4	–22.0	–21.4	–21.7±0.28	3.0	4.6	4.0±0.78
♂	2+	1			–22.8			7.2
♀		3	–22.1	–21.7	–21.8±0.23	5.4	5.7	5.6±0.15
?	1+	5	–22.8	–21.9	–22.5±0.34	4.8	7.8	6.4±1.11
?	0+	2	–24.2	–23.2	–23.7±0.71	7.5	9.0	8.3±1.06

^1^ ♂—males, ♀—females, ?—sex not determined.

The isotopic composition of carbon (δ^13^C) and nitrogen (δ^15^N) in bone collagen was determined by isotope mass spectrometry using a DELTA V Advantage isotope mass spectrometer (Thermo Fisher Scientific, Germany) equipped with a Flash 2000 elemental analyzer (devices provided by the shared use center TomCKP SB RAS) according to standard methods. The VPDB standard has been adopted as the international carbon standard. Gaseous N2 of atmospheric air is accepted as the international standard for nitrogen. Laboratory working gases for comparison, CO_2_ and N_2_, were calibrated according to the IAEA international standard sample—IAEA-600 Caffeine. The absolute measurement error of three consecutive measurements of the analyzed samples for δ^13^C did not exceed ±0.2‰, and for δ^15^N did not exceed ±0.4‰.

The values of the isotopic composition of carbon and nitrogen in the collagen of the bones of the Ural cave bear are presented in Table 1.

Mean δ^13^C values in groups of sub-adult and adult males and females in the North Urals differ by no more than 0.5‰, and δ^15^N values by no more than 0.9‰ (Table 1). In the Middle Urals, these differences for δ^13^С and δ^15^N are no more than 0.5‰ (Table 1). In general, the values of δ^13^С and δ^15^N for groups of subadult and adult individuals are close.

The differences in δ^13^С and δ^15^N values in groups of young individuals—2+, 1+, and 0+ years old—are much greater. The differences in δ^13^С values between males and females in the 2+ year group in the North Urals are 1.5‰, and in the Middle Urals – 1.0‰. The differences in the δ^15^N value are 1.9 and 1.6‰, respectively (Table 1). The differences in the δ^13^C value between groups 1+ and 0+ in the North Urals are 0.8‰, in the Middle Urals – 1.2‰, and the differences in the δ^15^N value are 2.0 and 1.9‰, respectively (Table 1).

The distribution of δ^13^С and δ^15^N values of age groups 1+ from the North and Middle Urals practically coincide and are almost completely outside the values for adult individuals (Fig. 1). The distribution of values for age groups 2+ of the North and Middle Urals coincide and partially overlap with the distribution of values for adult groups (Fig. 1). The values for age groups 2+ coincide with the values for age groups 1+. The values for individuals 0+ are outside the values for other age groups (Fig. 1).

**Fig. 1.**
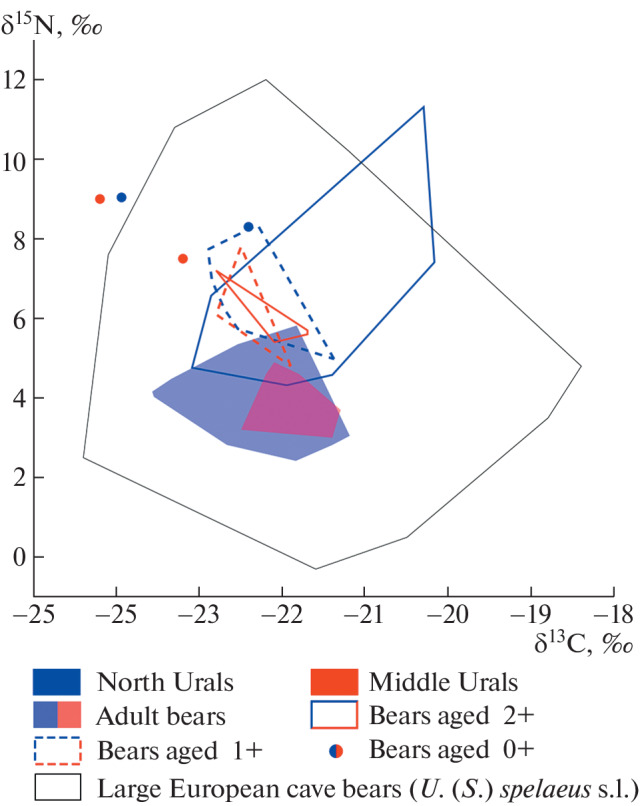
Distribution of δ^13^C and δ^15^N (‰) values in the collagen of the bones of the Ural cave bear (*U*. (*S*.) *kanivetz*) of the North (blue) and Middle (red) Urals of different ages and large European cave bears (*U*. (*S*.) *spelaeus* s.l.).

Differences between samples (with *n* > 4) were statistically assessed using the nonparametric Mann-Whitney test. The δ^13^C values do not differ statistically significantly between all samples. The δ^15^N values differ significantly between the 4 pairs of samples. In the North Urals, δ^15^N values are significantly (5% significance level) higher in the sample of age 1+ compared to the combined sample of males and females of age 2+ and in the combined sample of age 2+ compared to the combined sample of sub-adult (3+, 4+) males and females. In the Middle Urals, δ^15^N values are significantly (1% significance level) higher in the sample of age 1+ than in the combined sample of subadult (+3, +4) and adult (>4+) males and females. In the combined sample of adult (>4+) males and females from the Middle Urals, the δ^15^N values are significantly (5% significance level) higher than in a similar sample from the North Urals.

The data obtained show that almost all significant differences in δ^15^N values are observed between samples of juveniles (1+ and 2+) or between samples of juveniles (2+) and subadults and adults (3+ and older). Differences in δ^15^N values are determined by the proportion of protein foods in the diet [19]. High δ^15^N values in younger age groups (0+, 1+) are associated with their dairy diet, which contains large amounts of protein. At the age of 2+, animals begin to feed independently, but the signature from the previous period of milk feeding is retained in the bone collagen. One individual aged 2+ has a very high δ^15^N value—11.3 ‰. She may have continued to feed on milk into her second year of life.

There are reliably significant geographical differences between samples of adults. The δ^15^N values for bears in the Middle Urals (4.5‰) are higher than those for bears in the North Urals (3.9‰) by 0.6‰. These differences indicate a difference in diet, in our case, a different ratio of plant and meat foods. In the Middle Urals, the share of meat food in the nutritional structure was higher than in the North Urals, but this difference is less than the differences between trophic levels [20].

The distribution of δ^13^С and δ^15^N values in the collagen of the bones of the Ural cave bear almost completely coincides with the distribution of δ^13^С and δ^15^N in the collagen of the bones of cave bears (*U.* (*S*.) *spelaeus and U.* (*S*.) *eremus*) of Western and Central Europe [1–7] (Fig. 1). Outside the distribution there are three individuals, two of which are newborns (0+) and one young (2+) individual with an abnormally high δ^15^N value. This indicates the similarity of the diet of all three species of cave bears, i.e., the Ural (*U.* (*S*.) *kanivetz*) and the European (*U.* (*S.*) *spelaeus* and *U.* (*S*.) *eremus*).

Analysis of the data obtained shows the presence of significant age-related and geographical differences in the δ^15^N values in the collagen of the bones of the Ural cave bear of the North and Middle Urals. There is a significant trophic shift between age groups associated with the transition from milk feeding to independent feeding. There are geographical differences in the diet of adults in the North and Middle Urals. The diet of bears in the Middle Urals contained a higher proportion of meat food. Differences in δ^13^C values between age, gender and geographical samples are not significant. In general, adult and subadult males and females of the North and Middle Urals are at the same trophic level, because differences in δ^13^C and δ^15^N values (Table 1, Fig. 1) do not exceed the level of differences between different trophic levels [20]. The large cave bears of the Urals and Europe had a similar type of diet.
