# Erratum to: **Content of** ^**13**^ **С and** ^**15**^ **N Isotopes in Bone Collagen of Geographical, Age, and Sex Groups of the Ural Cave Bear (Mammalia, Carnivora, Ursidae,** ***Ursus (Spelaearctos) kanivetz*** **Verestchagin, 1973)**

**DOI:** 10.1134/S0012496623050095

**Published:** 2024-01-25

**Authors:** P. A. Kosintsev, K. Yu. Konovalova, G. V. Simonova

**Affiliations:** 1 Institute of Plant and Animal Ecology, Ural Branch, Russian Academy of Sciences, Yekaterinburg, Russia; 2 Institute of Monitoring of Climatic and Ecological Systems Siberian Branch Russian Academy of Sciences, Tomsk, Russia

The article “Content of ^13^ С and ^15^ N Isotopes in Bone Collagen of Geographical, Age, and Sex Groups of the Ural Cave Bear (Mammalia, Carnivora, Ursidae, *Ursus (Spelaearctos) kanivetz* Verestchagin, 1973),” written by P.A. Kosintsev, K.Yu. Konovalova, and G.V. Simonova, was originally published Online First in Springer-Link on November 11, 2023 without Open Access. After publication, the authors decided to make the article an Open Access publication. Therefore, the copyright of the article has been changed to © The Author(s) 2023 and the article is forthwith distributed under the terms of a Creative Commons Attribution 4.0 International License (http://creativecommons.org/licenses/by/4.0/, CC BY), which permits use, duplication, adaptation, distribution and reproduction of a work in any medium or format, as long as you cite the original author(s) and publication source, provide a link to the Creative Commons license, and indicate if changes were made.

